# Most common genotypes and risk factors for HCV in Gaza strip: a cross sectional study

**DOI:** 10.1186/1743-422X-6-105

**Published:** 2009-07-16

**Authors:** Basim M Ayesh, Sofia S Zourob, Salah Y Abu-Jadallah, Yonat Shemer-Avni

**Affiliations:** 1 Molecular Biology Lab, Central Laboratory-Ministry of Health, Gaza, Palestinian authority; 2 Biology Department-Islamic University of Gaza, Gaza, Palestinian authority; 3 The European Gaza Hospital Laboratory, Gaza, Palestinian authority; 4 Soroka Academic Medical Center, Faculty of Health Sciences, Clinical Virology Unit, Dept of Virology, Beer Sheva, Israel

## Abstract

**Background:**

The present work aims at determining HCV genotypes in patients with chronic HCV infection, in Gaza strip, Palestine. The most common risk factors for HCV transmission were also evaluated in conjunction with the genotyping data.

**Results:**

The study shows that there are only two major genotypes of HCV in Gaza Strip: Genotype 1 (subtypes 1a and 1b) collectively contribute to 28.3% of the cases, and genotype 4 (subtypes 4a and 4c/d) collectively contribute to 64.1% of the cases. Mixed infection with the two genotypes was also present among 7.6% of the cases. In this study a statistically significant relationship was established between the distribution of these genotypes and the patients' living place, traveling history, history of blood transfusion and history of surgical operations.

**Conclusion:**

The present study is the first to link HCV genotyping in Gaza strip with its possible roots of transmission. Traveling to endemic countries, especially Egypt; blood transfusion and surgical operations are major roots of HCV infection in Gaza strip. The results indicate that iatrogenic and nosocomial procedures may be responsible for the majority of HCV infections in Gaza strip.

## Background

Hepatitis C is caused by infection of liver cells with hepatitis C virus (HCV) leading to sever inflammation of the liver [1]. Most HCV infections persist, leading to chronic hepatitis, which can develop into chronic active hepatitis, liver cirrhosis and hepatocellular carcinoma [2].

The number of people infected with HCV has recently reached epidemic proportions and became a major global health issue. Over 170 million are infected worldwide [3]. The latest published HCV prevalence among Palestinian blood donors, in 2005, was 0.2% with an incidence rate of 5.2 per 100,000 [4]. Egypt, the closest neighbor to Gaza strip and its gateway to the world, has possibly the highest HCV prevalence in the world. A recent study showed that HCV antibody prevalence in a rural Egyptian area was 18.5%, reaching 45% in males over 40 years, and 30% in females over 50 years [5]. Approximately 20% of Egyptian blood donors are anti-HCV positive, and the strong homogeneity of HCV subtypes found in Egypt (mostly 4a) suggests an epidemic spread of HCV [6]. Other med-eastern countries such as Saudi Arabia, Syria and Jordan have HCV prevalence rates of 2.5%, 1% and 1.7% respectively [7-9]. In Israel, the reported prevalence of HCV infection is 0.5% [10].

HCV is known to have a marked genetic heterogeneity with an estimated nucleotide substitution rate of between 1.44 × 10^-3 ^and 1.92 × 10^-3 ^substitutions per site per year [11]. Accumulation of these nucleotide substitutions has resulted in the evolution of at least six major HCV genotypes (1–6) and more than 50 subtypes (designated by an alphabetic letter) that differ by as much as 30%–50% of their genome sequences [12]. Prevalence of the different HCV genotypes varies according to the geographic region [13,14].

There is evidence associating certain genotypes of HCV with more sever hepatic pathology or quicker disease progression [13,15,16]. HCV genotype is a major predictive factor of the treatment outcome, and hence has a strong influence on the choice of treatment [17,18]. If taken in conjunction with other factors important in therapy, HCV genotyping will reduce the expenses for HCV patient's treatment when the duration of the therapy is optimized, and thus allowing a larger number of patients to be considered. This is especially important in a place like Gaza strip that suffers from limited financial recourses. Currently, there is almost no available data about the most prevalent HCV genotypes in the Palestinian authority territories. Moreover, the clinical value of HCV genotyping in HCV treatment is underestimated by Palestinian physicians. A study conducted in 1998 showed that the most common genotype found in Gaza strip is type 4 [19]. The results of the study suggested that HCV root of transmission to Gaza strip is mainly from Egypt. This study however, was carried out on a small sample of a limited population, mostly blood donors, of Gaza strip.

In this study, we determined the distribution of HCV genotypes among chronically infected HCV patients in Gaza strip. We also used the data obtained from HCV genotyping and the data collected by a questionnaire to evaluate the possible role of some risk factors for HCV incidence as well as to uncover possible origin of transmission of the virus in Gaza strip.

## Results

### RT-PCR Amplification

Among the 100 samples tested by RT-PCR, 92 were found positive and gave the characteristic 298 bps band on the gel. Sensitivity of the test (≤50 copies/ml) was evaluated by testing samples with various levels of viremia. Specificity of the PCR test was confirmed by sequencing. The quality of PCR reactions and the lack of contamination were assessed by the inclusion of a negative (H_2_O) control which didn't show any PCR amplification in all experiments.

### HCV genotypes and subtypes in Gaza Strip

Only two major genotypes of HCV in Gaza Strip were detected (table 1): Genotype 1 (subtypes 1a and 1b), collectively contributed to 28.3% of the cases (n = 26), and genotype 4 (subtypes 4a and 4c/d), collectively contributed to 64.1% of the cases (n = 59). Mixed infection with genotypes 1 and 4 was also detected among 7.6% of the cases (n = 7).

**Table 1:** Frequencies of HCV genotypes in the 92 patients studied in Gaza Strip

**Genotype**	**N**	**%**
***Genotype 1 and its subtypes***	**26**	**28.3**
*1a*	17	18.5
*1b*	9	9.8
***Genotype 4 and its subtypes***	**59**	**64.1**
*4a*	40	43.5
*4c*/*d*	19	20.6
***Mixed Genotypes (1+4)***	**7**	**7.6**

***Total***	**92**	**100**

Because of the small number of patients classified under most of the detected subtypes, we collectively combined genotype 1 and its subtype under the same category; genotype 4 and its subtypes under a second category and the mixed infections under a third.

### Genotype and age of patient

The mean study population age was 47.55 ± 17.1 years. No statistically significant difference was detected between the mean age of the genotype groups. However, it is noteworthy to mention that 71.4% of mixed genotype infections are elder patients, over 60 years, compared to 15.4% for genotype 1 patients (P = 0.01, odds ratio = 13.75).

### Genotype and place of living

The majority of the study population (63%) were from the south of Gaza Strip and 34 (37%) were from the north. The majority of southern patients were infected with genotype 4 (72.4%) compared to (50.0%) of the Northern patients (p = 0.035) (Table 2).

**Table 2:** distribution of HCV genotypes among different study categories

			**Genotype**			**P-Value**
				
			**1**	**4**	**1+4**	
**Place of living**		**South**	11	42	5	0.035
			
		**North**	15	17	2	
			
		**Total**	26	59	7	

**Traveling**	**No**	**None**	12	22	1	
	
	**Before**	**Egypt**	1	25	2	0.000
			
		**Others**	3	2	2	
			
		**Egypt & others**	3	1	0	
			
		**Total**	7	28	4	
	
	**After**	**Egypt**	1	0	0	
	
	**Don't Know**	**Egypt**	4	7	1	0.742
			
		**Others**	1	1	0	
			
		**Egypt & others**	1	1	1	
			
		**Total**	6	9	2	

**Blood Transfusion**	**No**	**None**	15	35	2	
	
	**Before**	**Gaza**	10	11	4	0.017
			
		**Egypt**	0	13	1	
			
		**Jordan**	1	0	0	
			
		**Total**	11	24	5	

**Surgery**	**No**	**None**	12	33	3	
	
	**Before**	**Local**	8	7	2	0.014
			
		**Egypt**	0	13	0	
			
		**Others**	0	1	1	
			
		**Local & others**	2	1	0	
			
		**Egypt & others**	1	1	1	
			
		**Total**	11	23	4	
	
	**After**	**Local**	2	0	0	0.083

		**Egypt**	0	1	0	
			
		**Total**	2	1	0	
	
	**Don't Know**	**Local**	1	2	0	

**ALT level**		**≤ 40 IU **	7	39	5	0.002
			
		**> 40 IU **	19	20	2	
			
		Total	26	59	7	

If we look at the distribution of genotype 4 alone, we find that 71.2% of patients infected with this genotype are located in the Southern part of Gaza Strip compared to 28.8% in the Northern part. This difference is statically significant (P = 0.01; odds ratio = 3.29). On the other hand, type 1 is almost equally distributed among Southern patients (42.3%) and Northern patients (57.7%).

### Genotype and traveling history

Patients with a history of traveling abroad (n = 57) are almost twice those with no traveling history (n = 35). Thirty nine patients (68.4%) traveled before being infected (Table 2). Only one patient traveled after he was infected, while 17 patients (29.8%) couldn't recall if they traveled before or after the infection.

If we consider only patients with a traveling history before infection, we find that 71.8% have traveled to Egypt; 17.9% to other countries, like Jordan, Saudi Arabia and 10.2% to Egypt and others. Among those who traveled to Egypt before infection, significantly more patients 89.3% are infected with type 4 and its subtypes compared to the other genotypes (P value = 0.000).

### Genotype and history of blood transfusion

All of the 40 patients with a history of blood transfusion (43.5%), got their blood transfusion before being infected with HCV. Five out of 7 patients with mixed genotypes (71.4%) have a history of blood transfusion (table 2).

A significant relation could be established between the genotypes and place of transfusion (P value = 0.017). Thirteen out of 14 patients (92.9%) who had a transfusion in Egypt are infected with type 4. No preference however was seen between genotypes 1 (40%) or genotypes 4 (44%) for patients who had a transfusion in Gaza Strip (n = 25). Only one patient was transfused in Jordan and he is infected by genotype 1.

### Genotype and history of surgical operations

A total of 45 patients had a history of medical surgery (48.9%). Most of them (86.7%) were operated before HCV infection and only 6.7% were infected after surgery (Table 2). A significant relation could be established between HCV genotype and place of surgery (P-value = 0.014). All patients who had their surgeries in Egypt for example (n = 13) have type 4 infection, while slightly more patients with type 1 (47.1%) than type 4 (41.2%) had their surgeries in Gaza.

### Genotype and serum ALT level

A significant relation could be established between the number of patients with ALT values above or below 40 IU mL^-1 ^(the normal upper limit) and the genotype of the patient (P-value = 0.002). For example, 73.1% of the patients infected with type 1 had ALT values above 40 IU mL^-1^. On the other hand 66% of type 4 patients, and 71.4% of mixed-genotypes patients had ALT values lower than 40 IU mL^-1 ^(table 2).

## Discussion

HCV genotyping may shed light on its evolution, source of outbreaks, and risk factors. It may be used to identify the source of infection in cases of patient-to-patient transmission and is also useful in the study of other modes such as vertical (mother to baby), sexual transmission and needle stick injury [20-22].

Results of this study indicate that there are only two major genotypes of HCV among chronic carriers in Gaza Strip: Genotype 1 and its subtypes 1a and 1b, and genotype 4 and its subtypes 4a and 4c/d. Mixed infection with the two genotypes was also detected. HCV genotype 4 and its subtypes are the predominant genotypes in Gaza strip (64.1%).

Genotypes 1 and 4 were previously reported to be more pathogenic and considered more difficult to treat. The rate of progression to chronicity after acute exposure to HCV is significantly higher in patients exposed to HCV genotypes 1b and 4 infections than in patients exposed to other genotypes [14,23]. Patients with HCV genotype 1a or 1b have more severe liver disease and lower rates of response to interferon therapy than patients with HCV genotype 2a or 2b [24]. Recent clinical trials show that success of HCV genotype 4 therapy is dependent on the applied protocol. This genotype doesn't seem difficult to treat when Pegylated IFN and ribavirin combination therapy is applied, as the response to treatment may be at an intermediate level compared with genotype 1 and genotypes 2 or 3 [25]. Moreover patients infected with type 1 and 4 had a higher viral load than patients infected with other genotypes [26].

The two major HCV genotypes (1 and 4) were found to be significantly distributed among patients from the south and north of Gaza strip (P-value = 0.035). Genotype 4 and its subtypes are concentrated in the south of Gaza strip (71.2%), while genotype 1 is slightly more represented in the north (57.7%) than in the south (42.3%).

It is well documented that genotype 4 and its subtypes especially 4a are the predominant genotypes in Egypt, the southern border of Gaza strip and its only gateway to other parts of the world. Many Gaza residents, especially from the south, have historical relationships with the Egyptian people as members of many families were divided between Gaza and Egypt, while keeping strong social links with frequent visits from both sides. On the other hand, Israel with the most predominant genotype 1 and its subtypes borders Gaza strip from the north. Moreover, the Egyptians ruled Gaza strip in the period between 1948 and 1967 and Israel occupied Gaza at 1967 and controlled the Palestinian lives until the establishment of the Palestinian National Authority. Based on this information, the genotypes distribution in the south and north of Gaza and each of Egypt and Israel may be correlated.

Traveling to endemic areas is associated with increased risk of HCV infection [27]. Our results show that HCV patients who have traveled to a foreign country (62.0%) are almost twice those who haven't (38.0%). The majority of patients with a traveling history (68.4%) traveled before they were infected. Only one patient reported that he traveled after he was infected, and 17 patients (29.8%) did not know if they traveled before or after the infection.

Egypt was the most frequently visited country (72%of those with traveling history). Egypt shows the highest HCV prevalence in the world [14,28]. The majority of patients who traveled to Egypt before infection (89.3%) have genotype 4.

Surgery is another risk factor for nosocomial HCV transmission in a health care setting [29,30]. Our results show that almost half of the patients participating in the study (48.9%) had a surgical operation, of which 86.7% had their surgery before HCV infection. Furthermore, there was a significant correlation between the place of surgery and HCV genotype (P value = 0.014). All patients who had their surgeries in Egypt had genotype 4. This suggests that those patients might have got infected during surgery. On the other hand, slightly more patients with genotype 1 (47.1%) than genotype 4 (41.2%) had their surgeries in Gaza.

A significant relation was established between the number of patients with ALT values above or below 40 IU mL^-1 ^(the normal upper limit) and genotype of the patient (P-value = 0.002). Most of patients infected with genotype 1 (73.1%) have ALT values above 40 IU mL^-1^. On the other hand most of patients with genotype 4 (66%), and mixed genotypes (71.4%) had ALT values lower than 40 IU mL^-1^. These results confirmed earlier reports showing that patients infected with genotype 1 are more susceptible to liver complications, hepatocellular carcinoma, and decompensate liver disease, than those infected with genotype 4 [24].

During blood transfusion, there is high risk of getting infected with HCV, especially when laboratory screening techniques are absent or fail to detect HCV infection in a fraction of blood donations. This makes HCV responsible for about 90%–95% of transfusion-associated hepatitis cases [31,32].

Our results show that all patients with a history of blood transfusion (43.5%) got their blood transfusion before being infected with HCV. Blood transfusion gives a higher chance for infection with mixed genotypes than with either type 1 or type 4 alone. Five out of 7 patients with mixed genotypes (71.4%) have a history of blood transfusion.

A significant relation was established between HCV genotypes and the place of transfusion (P-value = 0.017). This relation is clear in the case of Egypt, as 13 out of 14 patients (92.9%) who had a transfusion in Egypt are infected by the genotype 4, the dominant genotypes in Egypt. No preference however could be seen between genotypes 1 (40%) or 4 (44%) for patients who had a transfusion in Gaza Strip. Double infection is present in 5 of 40 patients (12.5%) who had received blood. Those patients were exposed to repeated blood transfusion or infected by another rout of HCV transmission. Like in surgery, these data are supported by sequence comparison (not shown). One patient who had surgery in Egypt was infected by genotype 1a, a rare genotype in Egypt. However, this patient had previously a blood transfusion in Gaza, from which he might have gotten his infection. Screening for HCV in blood banks is a mandatory procedure in Gaza and partly in Egypt and a high risk of HCV infection among egyptian blood donors was previously reported [33].

## Conclusion

Our results show that there are only two major circulating genotypes of HCV in Gaza Strip: Genotype 1 (subtypes 1a and 1b); and genotype 4 (subtypes 4a and 4c/d). Mixed infection with the two genotypes was also seen. The results also show that traveling to endemic countries (particularly Egypt), blood transfusion and surgical operations are the major roots of HCV infection in Gaza strip. Therefore, iatrogenic and nosocomial procedures may be responsible for the majority of HCV infections in Gaza strip although other roots of transmission must be present as 14 patients (15.2%) were excluded from any of these categories.

## Materials and methods

### Patients and Samples collection

The present study is a descriptive study for genotyping HCV isolates from Gaza strip. The study population was chosen based on availability from patients attending the two major hepatology departments in a six-month period (100 patients). Male and female subjects who are chronically infected with HCV (based on previous diagnosis either by ELISA or RT-PCR) were included in the study. Only Patients positive for HCV RNA were included in sequencing and genotype determination (91 patients). Peripheral blood samples were collected, in plain tubes from 55 patients attending *the European Gaza hospital *(south of Gaza strip), and 45 patients attending *Al-Shiffa hospital *and *Al Remal clinic *(north of Gaza strip). Fifteen of these samples were collected from dialysis patients. Serum samples were prepared within no more than 4 hours after withdrawal and were immediately stored in sterile DNase- and RNase-free, tightly caped tubes, at -70°C.

The procedures of the study were approved by the local Helsinki committee according to the World Medical Association Declaration of Helsinki [34] and a written consent was obtained from each patient. A close-ended, dichotomous and multiple-choice based questionnaire was designed and completed based on patients' interview.

### Biochemistry

ALT was analyzed for all samples using Alcyon auto-analyser and commercially available kit (DiaSys Diagnostic System GmbH, Germany) according to the manufacturer instructions. The normal range for ALT was considered from 0 to 40 IU mL^-1^.

### Primers sequences

The forward primer: CCCTGTGAGGAACT***W***CTGTCTTCACGC (***W ****is A or T*) and the reverse primer: GGTGCACGGTCTACGAGACCT were designed to specifically bind the region between -299 and -1 of HCV genome 5'UTR respectively [35]. This region is highly conserved among the different genotypes. Sequence degeneracy was included to allow annealing to the various genotypes.

### Viral RNA Extraction and HCV RT-PCR amplification

Viral RNA was extracted from 140 μl serum samples using the QIAamp viral RNA Extraction kit according to the manufactures recommendations (Qiagen, Germany). Both cDNA synthesis and PCR amplification of target sequences were performed in a single tube using the QIAGEN one step RT-PCR kit (Qiagen, Germany). The reactions were carried out in 25 μl reaction volumes using 10 μl RNA in the presence of 0.6 μM of each primer, 400 μM of each dNTP and 5 units RNase inhibitor. The reaction cycling conditions were: one cycle at 50°C for 30 minutes and one cycle at 95°C for 15 minutes followed by 40 cycles of 95°C for one minute, 55°C for one minute and 72°C for one minute. Finally the reactions were allowed to complete at 72°C for 10 minutes and held at 4°C. The products were analyzed on 2% agarose gel and stained with ethedium bromide. The PCR products were purified from the gel using the *Qiaquick Gel Extraction Kit *according to the manufacture instructions (Qiagen, Germany).

### HCV genotyping and sequence analysis

Although a variety of methods have been used for HCV genotyping, sequencing of an informative region of its genome (the 5'UTR) remains the golden standard. The 5'UTR is highly conserved, and therefore is very suitable for both RT-PCR detection and genotyping of HCV. Sequencing and genotype determination of the purified PCR products were conducted by Hy-labs (Park Tamar, Rehovot), using the Big Dye Terminator Cycle Sequencing kit and the analysis software for downstream analysis of sequences (ABI, USA).

Polygenetic analyses were performed on the 5'-UTR of HCV (175 bp, position 11–185 gb|DQ418782.1|. Pairwise analysis was performed using multiple sequence alignment. The ClustalW program [36] was used to determine the genetic distance; divergence of nucleotide substitutions per 100 nucleotide sites and neighbor joining slanted phylogram tree analysis. Comparison between means of nucleotide variability was carried out using Student's t-test for independent samples. Results were considered significant at P < 0.05.

### Statistical analysis

The questioner results were transformed into numerical scoring system, and the statistical analysis was carried out (by SPSS and Epi-info), using frequencies and cross tabulation between dependant and independent variables. Chi square test and T test were used and P-values of < 0.05 were considered statistically significant.

## Competing interests

The authors declare that they have no competing interests.

## Authors' contributions

BMA participated in the design of the study; designed and supervised on the molecular genetic studies and the statistical analysis and drafted the manuscript. SZ carried out and personally financed the molecular genetic studies and statistical analysis. AYAJ helped in supervision on the theoretical and practical work. YSA coordinated and financed the sequencing and genotyping, and revised the draft manuscript.
